# Relationship between the Clinical Characteristics in Patients with Neuromyelitis Optica Spectrum Disorders and Clinical Immune Indicators: A Retrospective Study

**DOI:** 10.3390/brainsci12030372

**Published:** 2022-03-11

**Authors:** Linjun Cai, Ziyan Shi, Hongxi Chen, Qin Du, Ying Zhang, Zhengyang Zhao, Jiancheng Wang, Yanling Lang, Lingyao Kong, Hongyu Zhou

**Affiliations:** Department of Neurology, West China Hospital, Sichuan University, Chengdu 610041, China; ccdclj@126.com (L.C.); shiziyan129@163.com (Z.S.); chen_hongxi@126.com (H.C.); 18202814276@163.com (Q.D.); zhangying94322@163.com (Y.Z.); zhengyang@outlook.com (Z.Z.); wangjiancheng101@gmail.com (J.W.); 18381312397@163.com (Y.L.); lykongv@163.com (L.K.)

**Keywords:** neuromyelitis optica spectrum disorders, T lymphocyte, complement, immunoglobulin, treatment, motor disability

## Abstract

Objective: T lymphocytes, complement, and immunoglobulin play an important role in neuromyelitis optica spectrum disorders (NMOSD). As common clinical examination indicators, they have been used as routine indicators in many hospitals, which is convenient for being carried out in clinical work, but there are few articles of guiding significance for clinical practice. The purpose of this study was to study the relationship between commonly used immune indicators and clinical characteristics in patients with NMOSD. Methods: We compared clinical characteristics and clinical immune indicators in 258 patients with NMOSD and 200 healthy controls (HCs). We used multiple linear regression to study the relationship between immunotherapy, disease phase, sex, age, AQP4-IgG, and immune indicators. In addition, lymphocyte subsets were compared before and after immunotherapy in 24 of the 258 patients. We explored the influencing factors and predictors of severe motor disability. Results: The percentages of CD3 ratio (71.4% vs. 73.8%, *p* = 0.013), CD4 ratio (38.8% vs. 42.2%, *p* < 0.001), and CD4/CD8 ratio (1.43 vs. 1.66, *p* < 0.001) in NMOSD patients were significantly lower than those in the HC group. In addition, complement C4 (0.177 g/L vs. 0.221 g/L, *p* < 0.001) and peripheral blood IgG (10.95 g/L vs. 11.80 g/L, *p* = 0.026) in NMOSD patients were significantly lower than those in the HC group. CD3 percentage was correlated with blood collection age and disease stage; CD8 percentage was correlated with blood collection age, disease stage, and treatment; CD4/CD8 percentage was correlated with blood collection age and treatment; complement C4 was correlated with blood collection age and sex; and IgG was correlated with disease stage and treatment. Twenty-four patients before and after treatment showed that the percentages of CD3 ratio (74.8% vs. 66.7%, *p* = 0.001) and CD8 ratio (32.4% vs. 26.2%, *p* < 0.001) after treatment in NMOSD patients were significantly increased, and the percentage of CD3 before treatment was moderately negatively correlated with ARR (r = −0.507, *p* = 0.011). Binary logistic regression analysis showed that peripheral blood complement C3 is a serious influencing factor for severe motor disability (EDSS score ≥ 6 points). Peripheral blood complement C3 and C4 are predictors of severe motor disability (*p* < 0.05). Conclusion: Our results suggest that peripheral blood T lymphocytes, C3, C4 and immunoglobulin are convenient and routine clinical indicators that are convenient for implementation in clinical work. They have certain reference values for disease staging, recurrence, drug efficacy, and motor disability. They have improved our understanding of clinical immune indicators for NMOSD patients, but whether they can be used as biomarkers for clinical prognosis remains to be further studied.

## 1. Introduction

Neuromyelitis optica spectrum disorders (NMOSD) is considered an autoimmune and inflammatory disease of the central nervous system (CNS) that primarily attacks the optic nerve and spinal cord [1,2]. In the past, most studies believed that NMOSD is a central nervous system inflammation mainly mediated by B cells, but the role of T cells in NMOSD has also received attention in recent years. Most patients with NMOSD produce an IgG1-subclass [3] autoantibody called NMO-IgG against aquaporin-4 (AQP4-IgG) via a T cell-dependent mechanism [4,5]. The binding of AQP4-IgG to astrocytic AQP4 results in the activation of the classical complement cascade and infiltration of granulocytes and lymphocytes that collectively damage neural tissue. Disruption of the blood–brain barrier (BBB) is the first critical step in the pathogenesis of NMOSD, allowing the influx of humoral factors, including autoantibodies, through the dysfunctional barrier and infiltration of inflammatory cells [6]. In these ways, T lymphocytes, complement, and immunoglobulin play an important role in the occurrence and development of NMOSD.

T cells are the most numerous and most complex type of lymphocytes, and they are mainly involved in cellular immunity and auxiliary humoral immunity. CD3 is an intrinsic molecule on the surface of mature T cells that binds to T cell surface receptors to form a TCR-CD3 complex that participates in antigen recognition, and CD3+ T cells reflect the immune function status of the human body. According to different functions and molecular phenotypes, mature T cells can be divided into two subgroups: CD4+ T cells and CD8+ T cells. The pathogenesis of NMOSD appears to involve Th1 and Th17 cells belonging to the CD4+ subset of T cells [7,8,9,10,11,12,13,14]. Our previous research using flow cytometry technology found significantly reduced CD8+ TN and increased CD8+ TE/M in both NMOSD and MS, while decreased CD8+ TMP was only observed in NMOSD. Patients treated with immunotherapy were associated with increased CD8+ TN and decreased CD8+ TE/M in NMOSD. These results have also implicated CD8+ T cell subsets in promoting the occurrence and development of the disease [15]. Different drug treatments have different effects on T lymphocytes. It has been reported that peripheral blood T lymphocyte subsets decreased in NMOSD patients receiving rituximab for up to 3 months, while the levels were similar to those at baseline after 4–6 months of rituximab therapy [16]. This shows that the duration of immunotherapy can affect T lymphocyte subsets. A small number of studies have reported the relationship between complement, immunoglobulin and disease activity, and EDSS score [17,18,19,20]. However, the abovementioned detection methods are less convenient. We hope that there will be indicators that can be found in clinical laboratory tests that can identify changes in the patient’s condition and have a higher feasibility and lower prices.

Peripheral blood T lymphocyte subsets, complement, and immunoglobulin are commonly used immune indicators in most hospitals in China. The inspection methods are convenient and easy to widely carry out in clinical work. We retrospectively analyzed the immune indicators of patients with NMOSD from our database and further explored their relationship with clinical characteristics, which may provide references for follow-up clinical practice.

## 2. Methods

### 2.1. Patient Selection and Data Collection

We retrospectively collected patient data from January 2009 to January 2021 in our database. The inclusion criteria were as follows: 1. Comply with the NMOSD diagnostic criteria established by the IPND in 2015 [21]; 2. Complete clinical data (cases with complete data in our database: complete collection of the annual recurrence rate of patients, imaging examinations, patient treatment plans, disability progression score, etc.); 3. All patients completed AQP4-IgG, MOG-IgGand T lymphocyte tests. We excluded patients with positive myelin oligodendrocyte glycoprotein immunoglobulin G (MOG-IgG)-positive, lost to follow-up, incomplete clinical data, severe or infectious comorbidities, immune-related indicators suggesting the coexistence of other autoimmune diseases, and patients with no EDSS score assessed during the first blood collection. Among them, 24 NMOSD patients had two blood samples in remission (before and after treatment). Two hundred age- and sex-matched healthy controls (HCs) were matched for comparison. The healthy controls were from the physical examination center of our hospital, and none of them had chronic diseases such as other neurological diseases, other autoimmune diseases, cardiovascular and cerebrovascular diseases, severe infections, tumors, or severe liver and kidney insufficiency.

Clinical data were collected through the hospital electronic system and follow-up database at our center. Data included sex, age, age at onset, annualized relapse rate (ARR) by the time of peripheral blood collection, disease duration, initial onset syndromes, proportion of T lymphocyte subsets in peripheral blood, complement C3 and complement C4 in peripheral blood, immunoglobulin G, treatment drugs, treatment time, EDSS score at peripheral blood collection, and EDSS score at the last follow-up. We study the correlation between the clinical data and immune indicators. Treatment drugs refer to long term immunosuppressive treatments or oral low doses of glucocorticoids (<1 mg/kg/d). Clinical relapse of NMOSD was defined as the onset of new neurological symptoms within 30 days after the last NMOSD episode, as long as the new symptoms lasted at least 24 h, were not related to fever or infection, and were accompanied by new objective neurological signs or imaging evidence [22]. Each patient was evaluated many times for the face-to-face follow-up EDSS score after blood sample collection, and EDSS scores were evaluated by two neurologists.

Blood samples of all NMOSD patients were collected in the outpatient department of our hospital or on the first day of hospitalization. A total of 58.1% of NMOSD patients were enrolled after treatment, and 41.9% of NMOSD patients were enrolled before treatment. Patients receiving treatment were defined as taking oral glucocorticoids within 1 month before blood collection or taking immunosuppressive agents within 3 months. In addition, the effects of receiving different immunosuppressants on immune indicators were compared. To eliminate the effect of high-dose corticosteroids on immune indicators, we compared the T cells proportion, C3, C4, and IgG of 49 NMOSD patients who received high-dose corticosteroids in the acute phase and 119 patients who did not receive high-dose corticosteroids in the acute phase.

Patients were subgrouped according to whether they were in the acute phase of NMOSD at the time of blood collection, defined as within one month of onset or recurrence of NMOSD [23], or in the remission phase, defined as disease that had remained stable for more than one month before blood collection. We explored the influencing factors of severe motor disability and the diagnostic value of immune indicators for severe motor disability. Severe motor disability was defined as an EDSS score ≥6 points. An EDSS score of 6 was defined as walking without assistance (cane, crutch, or brace), walking distance less than 100 m or walking distance with unilateral assistance ≥50 m, or walking distance with bilateral assistance ≥120 m.

The study was approved by the Medical Ethics Committee of the West China Hospital of Sichuan University (2018–2-7). All participants signed a consent form allowing their blood to be drawn for analysis and their anonymous data to be published for study purposes.

### 2.2. Laboratory Tests

Peripheral blood T lymphocyte subsets were detected by flow cytometry (FACSAria, BD, San Jose, CA, USA). The CD3, CD4, and CD8 molecules on the surface of lymphocytes were marked with fluorescent staining, and CD3+ T cells were identified. To further distinguish CD4+ T lymphocytes and CD8+ T lymphocytes, we counted the total number of T lymphocytes, CD4+ T lymphocytes, and CD8+ T lymphocytes, from which we calculated the CD4/CD8 ratio. To address this issue about batch differences in the fluorescence-activating cell sorter, we used the same batch compensation and isotype controls.

All patients underwent an AQP4-IgG antibody test using a commercial cell-based assay (EUROIMMUN, Luebeck, Germany). The human complement C3 and complement C4 complex EFSIA kit (China Beijing 4A Biotechnology Co., Ltd., Beijing, China) were used to detect complement C3 and C4 in the patient’s serum. The immunoturbidimetric assay kit was used to detect the peripheral blood immunoglobulin G (IgG) (China Nanjing Jidan Biotechnology Co., Ltd., Nanjing, China). At the same time, various factors related to the immune system or infection were checked to exclude the possibility of other autoimmune diseases or infectious diseases.

### 2.3. Statistical Analysis

Continuous data were checked for normal distribution using the Kolmogorov–Smirnov test. Categorical data are shown as numbers, and percentages and were compared between the two groups using chi-squared or Fisher’s exact tests. Normally continuous data were expressed as percentages (mean ± SD) and compared between groups using Student’s *t* test. Skewed data were expressed as medians (interquartile range) and compared between groups using the Mann–Whitney test. One-way ANOVA was used to compare multiple groups with Bonferroni correction. Multiple linear regression was used to find clinical influencing factors of immune indicators.

A paired sample t test was used to compare T lymphocyte subsets before and after treatment. Pearson correlation analysis or Spearman correlation analysis was used to evaluate the correlation between clinical immune indicators and ARR and EDSS scores. Binary logistic regression analysis was used to determine the influencing factors of severe motor disability. The best cut-off value of the ROC analysis immune index was drawn to diagnose severe motor disability in NMOSD patients.

All statistical analyses were performed using SPSS 23.0 (IBM, Chicago, IL, USA), and differences associated with a two-tailed *p* < 0.05 were considered statistically significant. All figures were generated using GraphPad Prism 8.0 software for Windows (San Diego, CA, USA).

## 3. Results

### 3.1. General Clinical Characteristics

**Figure 1:**
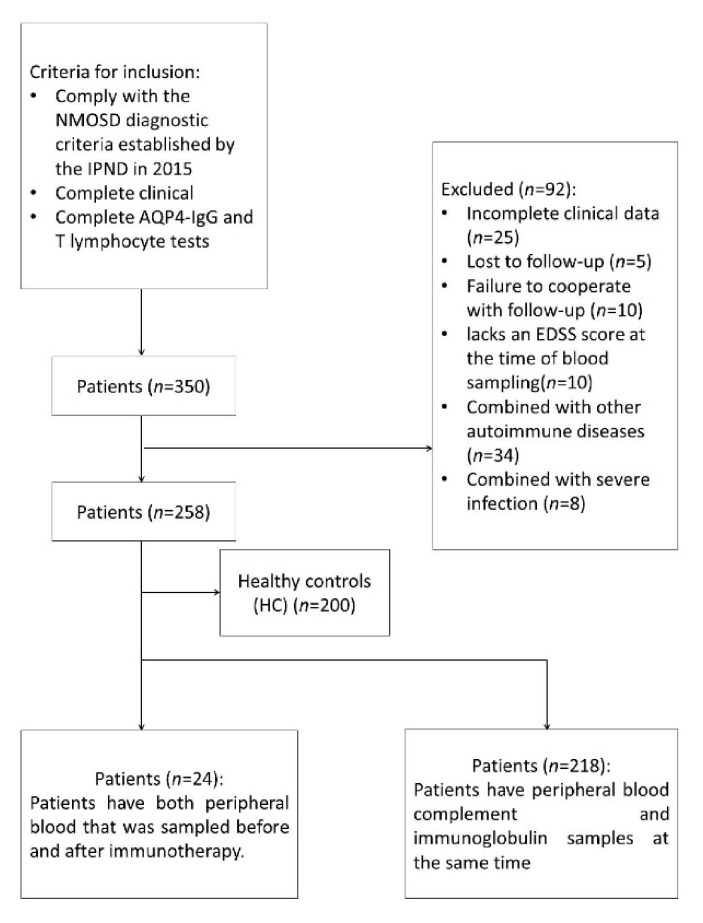
Flowchart.

According to the inclusion and exclusion criteria, 258 patients were ultimately screened from our database (Figure 1). Among them, there were 218 cases with peripheral blood complement and immunoglobulin G blood specimens. Among the included patients, the peripheral blood of 24 patients was sampled before and after immunotherapy. The average blood collection age of patients was 44.1 ± 14.5 years; they were predominantly female (90.3%), and their median duration of disease was 1.42 (5.33) years. There were 116 NMOSD patients with relapse after treatment, and 35 patients without relapse. There was no significant difference in T cells, C3, C4, or IgG between the two groups (*p* > 0.05) (Table 1). General clinical characteristics are shown in Table 1.

### 3.2. Relationship between Immune Indicators and Clinical Characteristics

As shown in Table 2, the percentages of CD3 ratio (71.4% vs. 73.8%, *p* = 0.013), CD4 ratio (38.8% vs. 42.2%, *p* < 0.001), and CD4/CD8 ratio (1.43 vs. 1.66, *p* < 0.001) in NMOSD patients were significantly lower than those in the HC group. In addition, complement C4 (0.177 g/L vs. 0.221 g/L, *p* < 0.001) and peripheral blood IgG (10.95 g/L vs. 11.80 g/L, *p* = 0.026) in NMOSD patients were significantly lower than those in the HC group.

To avoid interference between various factors, we used multiple linear regression to control for confounding factors. We included sex, blood collection age, AQP4-IgG serum antibody status, disease phase, and treatment as independent variables, and CD3 percentage, CD4 percentage, CD8 percentage, CD4/CD8 ratio, C3, C4, and IgG as dependent variables. Independent variables with *p* < 0.5 in the analysis were included in the multiple linear regression (Table 3). A multiple regression model with CD3 percentage as the dependent variable showed a correlation with blood collection age and disease phase, with an R2 of 0.058 for the final model. A multiple regression model with CD8 percentage as the dependent variable showed a correlation with blood collection age, disease phase, and treatment, with an R2 of 0.091 for the final model. A multiple regression model with a CD4/CD8 percentage as the dependent variable showed a correlation with blood collection age and treatment, with an R2 of 0.075 for the final model. A multiple regression model with C4 as the dependent variable showed a correlation with blood collection age and sex, with an R2 of 0.038 for the final model. A multiple regression model with IgG as the dependent variable shows a correlation with disease phase and treatment, with an R2 of 0.099 for the final model.

To evaluate the influence of patients’ drug treatment responses on immune indicators, we compared T cells proportion, C3, C4, and IgG of the patients who had relapses after treatment and the patients who had no relapses after treatment, and there was no difference between the two groups (*p* > 0.05). In addition, we compared the T cells proportion, C3, C4, and IgG of 49 NMOSD patients who received high-dose corticosteroids in the acute phase and 119 patients who did not receive corticosteroids in the acute phase, and there was no significant difference between the two groups (*p* > 0.05).

The percentage of CD3 and CD8 in patients treated with rituximab were significantly higher than those treated with MMF (78.3% vs. 68.5%, *p* = 0.012; 28.3% vs. 26.0, *p* = 0.042) (Table 4). The remaining immune indicators were not different among NMOSD patients who received different immunosuppressive treatments. (Table 4 and Table 5).

**Figure 2:**
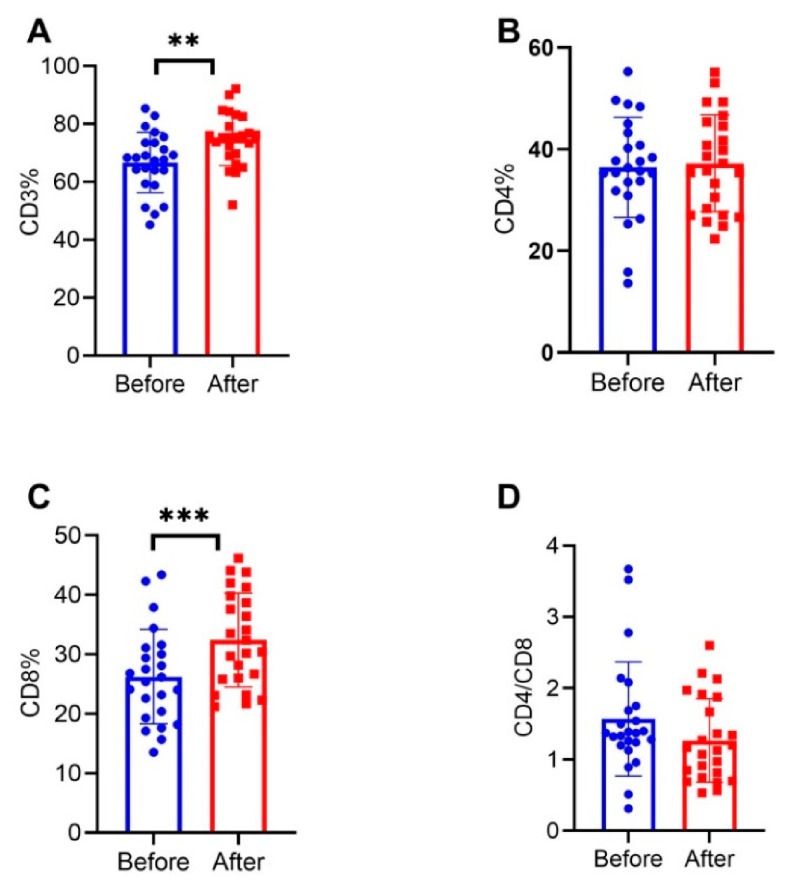
Comparison of T lymphocyte subsets before and after treatment in 24 patients with NMOSD (longitudinal) ** *p* < 0.01, *** *p* < 0.001. Blue circles represent T cells before immunosuppressive therapy. Red squares represent T cells after immunosuppressive therapy. (**A**) Comparison of CD3 percentage before and after treatment in 24 patients with NMOSD. (**B**) Comparison of CD4 percentage before and after treatment in 24 patients with NMOSD. (**C**) Comparison of CD8 percentage before and after treatment in 24 patients with NMOSD. (**D**) Comparison of CD4/CD8 ratio before and after treatment in 24 patients with NMOSD.

**Figure 3:**
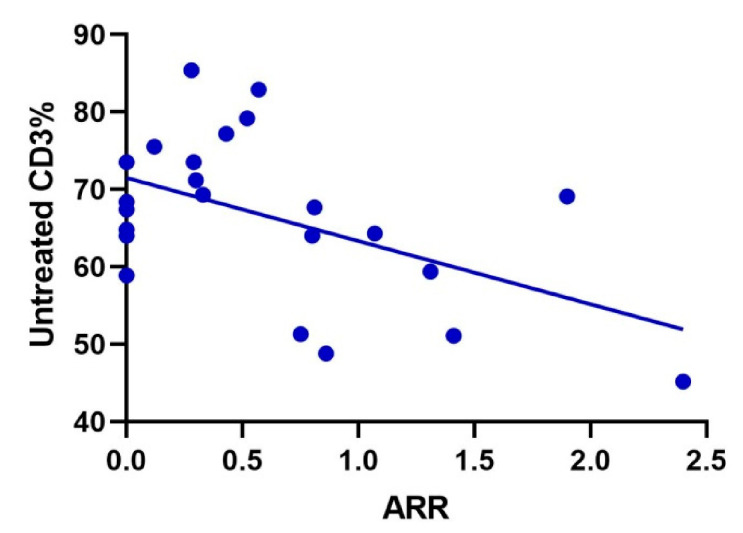
Correlation analysis of CD3 percentage before treatment and ARR in 24 patients with NMOSD.

Data from 24 patients were collected before and after treatment. The median treatment time was 19.0 (17.4) months. Compared with before treatment, the percentages of CD3 and CD8 in NMOSD patients after treatment were significantly higher (74.8% vs. 66.7%, *p* = 0.001 and 32.4% vs. 26.2%, *p* < 0.001, respectively), and the percentage of CD4 and the ratio of CD4/CD8 were not significantly different (*p* > 0.05) (Figure 2). The Pearson correlation analysis showed that the percentage of CD3 before treatment in NMOSD patients was moderately negatively correlated with ARR (r = −0.507, *p* = 0.011), and there was no correlation between T lymphocytes and EDSS score (*p* > 0.05) (Figure 3).

There was a significant negative correlation between the percentage of CD3 and ARR in 24 NMOSD patients before treatment (r = −0.507, *p* = 0.011). |r| > 0.7 indicates that there is a strong correlation between variables; 0.3–0.7 indicates that there is a moderate correlation between variables; and < 0.3 indicates that there is a weak correlation between variables.

We used severe motor disability (Y) in NMOSD patients as the dependent variable. Factors that may affect motor disability, such as sex, blood collection age, AQP4 antibody, disease course, spinal cord involvement segment, T lymphocytes, complement C3, complement C4, and IgG, were included in the univariate analysis. The variables with *p* < 0.1 in the univariate analysis were included in the binary logistic regression model for analysis. As shown in Table 5, blood collection age (OR [95% CI] = 1.080 [1.044–1.117], *p* = 0.000), sex (OR [95% CI] = 0.170 [0.041–0.701], *p* = 0.014), spinal cord involvement segment ≥ 3 (OR [95% CI] = 7.763 [2.202–27.370], *p* = 0.001), and complement C3 (OR [95% CI] = 22.598 [2.532–201.647], *p* = 0.005) were severe motor disability-influencing factors (Table 6).

**Figure 4:**
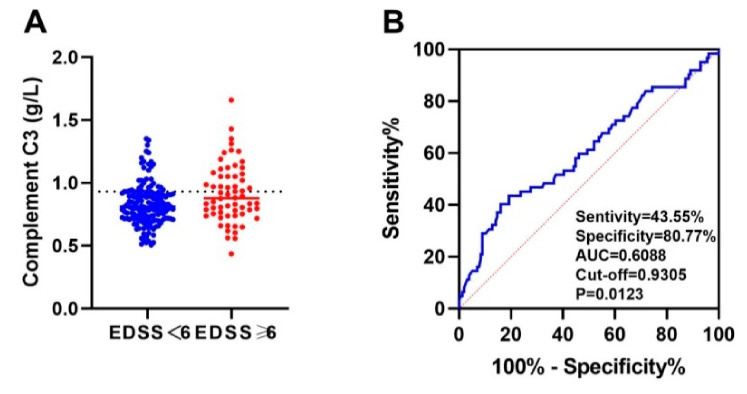
The ROC curve of serum complement C3 predicting severe motor disability in NMOSD patients. (**A**) Comparison of complement C3 between two groups of people with EDSS score <6 and ≥6. (**B**) The area under the curve (AUC) of peripheral blood complement C3 for predicting severe motor disability was 0.6088, and the best cut-off value was 0.9305 g/L. The sensitivity and specificity of complement C3 > 0.9305 g/L to predict severe motor disability were 43.55% and 80.77%, respectively.

**Figure 5:**
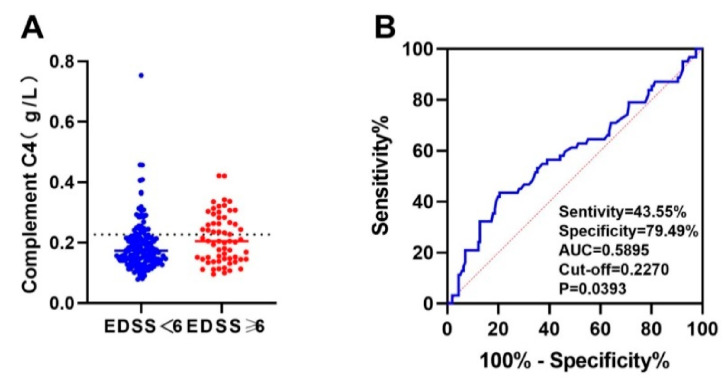
The ROC curve of serum complement C4 predicting severe motor disability in NMOSD patients. (**A**) Comparison of complement C4 between two groups of people with EDSS score <6 and ≥6. (**B**) The area under the curve (AUC) of complement C4 for predicting the prognosis of severe motor disability was 0.5895, and the best cut-off value was 0.2270 g/L. The sensitivity and specificity of complement C4 > 0.2270 g/L to predict severe motor disability were 43.55% and 79.49%, respectively.

We used ROC curve analysis and included all immune indicators. The results showed that peripheral blood complement C3 and C4 are predictors of severe motor disability (EDSS score ≥ 6 points) (*p* < 0.05). The area under the curve (AUC) of peripheral blood complement C3 for predicting severe motor disability was 0.6088, and the best cut-off value was 0.9305 g/L. The sensitivity and specificity of complement C3 > 0.9305 g/L to predict severe motor disability were 43.55% and 80.77%, respectively (Figure 4). The area under the curve (AUC) of complement C4 for predicting the prognosis of severe motor disability was 0.5895, and the best cut-off value was 0.2270 g/L. The sensitivity and specificity of complement C4 > 0.2270 g/L to predict severe motor disability were 43.55% and 79.49%, respectively (Figure 5).

## 4. Discussion

We found that immune indicators such as the CD3 percentage, CD4 percentage, CD4/CD8 ratio, complement C4, and IgG in NMOSD patients were lower than those in healthy people, which shows that the immune function of NMOSD patients is reduced. There is increasing evidence that different subgroups of T lymphocytes play important roles in the occurrence and development of NMOSD [15,24,25]. We found a correlation between T lymphocyte subsets and disease activity in patients with NMOSD in this study, and we found that immunotherapy was associated with an increase in T lymphocyte subsets. We also found evidence that CD3+ T cells before immunotherapy correlated with ARR. Complement C3 and C4 have a weaker predictive value in predicting motor disability. Our results provide a reference for future clinical practice.

The development of autoimmunity in the CNS is triggered by autoreactive T cells, which are activated and gain the ability to enter the CNS through the BBB [6]. Under normal circumstances, T lymphocytes maintain the body’s normal immunity by interacting with B lymphocytes, and their different subgroups are also in dynamic equilibrium [26]. CD4+ T cells and CD8+ T cells are the most important subgroups of T lymphocytes, and they are clinically significant in a variety of autoimmune diseases. In clinical work, the percentages and ratio of CD4+ T cells and CD8+ T cells can be used to determine whether the body’s immune function is disordered.

There are conflicting results in the study of T cell subpopulations for NMOSD. One study reported that the percentage of CD4 in 35 NMOSD patients was not different from that in healthy controls [27], while another study found that both Th17 and Th22 were higher in 21 NMOSD patients than in the control group [25], while some anti-inflammatory cells, such as regulatory T cells (Tregs), were found to be reduced in NMOSD. We speculate that anti-inflammatory cells may have a more obvious role in NMOSD patients. Regarding the changes in CD8+ T cells, a previous study by our team found that compared with healthy people, the proportion of naive CD8+ T cells in NMOSD patients was significantly reduced, and the proportion of effector/memory CD8+ T cells was significantly increased [15]. Lucchinetti et al. previously reported that NMOSD patients had a small amount of infiltration of CD3+ T and CD8+ T cells around the blood vessels [28]. However, we did not find differences in the total CD8 percentage from healthy people, although there were differences between subgroups. We speculate that the transfer of CD8+ T cells may be related to disease activity and treatment. In addition, our study also found that the peripheral blood CD3 percentage, CD4 percentage, and CD4/CD8 ratio of NMOSD patients were lower than those of healthy controls. Previous studies have found that CD8+ T cells can independently induce mild central nervous system diseases, and CD4+ T cells are the main driving force of CNS autoimmunity [29]. This result further confirmed that the reduction in CD4+ T lymphocytes may be a factor that causes the body’s weakened immunity. The apoptosis of CD4+ T lymphocytes in peripheral blood can lead to a decline in the body’s immune function by inhibiting the proliferation and activation of T lymphocytes or B lymphocytes [30]. We speculate that the possible causes of the decrease in CD4+ T cells in the periphery of NMOSD are as follows: 1. It is consumed in the acute phase; 2. The blood–brain barrier is destroyed and enters the central nervous system from the periphery; 3. The imbalance of peripheral lymphocytes [31].

Several studies have found that T lymphocytes are associated with disease activity. Animal experiments have shown that the direct injection of AQP4 antibody into the brain tissue of BBB-disrupted mice or rabbits does not cause destruction of astrocytes, but if aquaporin 4-specific T cells are involved, it will cause tissue damage [32]. This shows that T cells have a strong correlation with the activation of diseases. Tfh cells are a subpopulation of CD4+ T cells, and Li et al. found that the number of Tfh cells in the acute phase of NMOSD was greater than that in the remission phase [33]. However, another study found that the proportion of IL-6^+^ and IL-17^+^ Tfh cell subsets was higher in NMOSD patients than in healthy individuals and the frequency of both Tfh cell subsets was directly associated with disease activity [34]; however, how Tfh cells participate in the pathogenesis of NMOSD remains to be studied. In previous studies, we did not find that CD8+ T cells are correlated with disease activity [15]. In this research, we found that the percentages of CD3 and CD8 were correlated with disease stage, and the percentages of CD3 and CD8 were higher in NMOSD patients in remission after eliminating the interference of drugs on T cells. We speculate that CD3+ T and CD8+ T cells enter the central nervous system to participate in the acute immune response. More accurate conclusions need to be confirmed by further studies.

Age is an important factor affecting the prognosis of disease, and some studies have shown that naive CD4+ T cells are negatively correlated with age [35]. In our previous study, we found that both phenotypes of CD8+ T cells in NMOSD patients decreased linearly with age. In this research, we also found that age was also correlated with the percentages of CD3 and CD8, with older NMOSD patients having lower CD3 and CD8 percentages. The number of lymphoid hematopoietic stem cells decreases with age, which in turn leads to a decrease in the number of lymphocytes migrating to secondary lymphoid tissues and peripheral circulation. In addition, due to the degeneration of the thymus and the reduced function of mature lymphocytes in secondary lymphoid tissues, the production of primary lymphocytes is significantly reduced in elderly individuals [36,37], which may explain the lower T lymphocytes in patients with NMOSD as they get older.

Previous studies have reported that AQP4-IgG-positive patients have more abnormal CD4/CD8 ratios than AQP4-IgG-negative patients, and more of them have a lower ratio [38], but our results did not show this phenomenon. The AQP4-specific antibody in NMOSD serum requires the help of T cells. AQP4-specific CD4+ T cells participate in the occurrence of this adaptive humoral response, and we detected that the proportion of T lymphocytes is a total value. AQP4 autoreactive T cells account for only a small part of the total value, which requires further study by enlarging the sample size.

The most significant effect of glucocorticoids on immune function, especially on T cells, is an immunosuppressive effect. The change in the T lymphocyte ratio can reflect the effect of drug therapy to a certain extent. The EAE model showed that after receiving MMF treatment, the proportion of CD8+ T cells in the peripheral blood increased, and the proportion of CD4+ T cells and the ratio of CD4/CD8 decreased, which suggests that MMF may produce immunosuppressive effects by increasing CD8+ T cells [39]. In addition, it has been reported that the CD3+ T, CD4+ T, and CD8+ T lymphocytes of NMOSD patients receiving rituximab treatment for 0–3 months decreased, but there was no significant change after 4–6 months [16]. Previous studies in our center showed that naive CD8+ T cells in peripheral blood increased and effector/memory CD8+ T cells decreased after immunotherapy [15].

In our cross-sectional study, we found an increased percentage of CD8 in NMOSD patients after 6.5 months of immunotherapy. In addition, the percentages of CD3 and CD8 in NMOSD patients treated with rituximab were significantly higher than those in patients treated with MMF. Rituximab is a B cell-depleting agent. It is difficult to assess whether rituximab inhibits B cells and thus increases the proportion of T cells, or whether rituximab indirectly regulates T cell proliferation. To further explore the effect of immunotherapy on T lymphocyte subsets, we conducted a small longitudinal study. The 24 patients received immunotherapy for a median time of approximately 19 months, and it was found that the percentages of CD3 and CD8 increased after treatment. This is consistent with the report of the EAE model and our cross-sectional study. There are many subgroups of CD8+ T lymphocytes, and there are differences between different subgroups. Therefore, we speculate that immunotherapy may work by increasing the ratio of CD8+ T cells or reducing the ratio of CD4+ T cells. Immunotherapy has a more significant effect on the increase in CD8+ T cell subsets. The increase in the total CD3 and CD8 percentages after treatment also explains the increase in the CD3 and CD8 percentages after drug treatment in remission.

In addition, we also found that the CD3 percentage of patients before treatment was negatively correlated with ARR. We speculated that there might be a correlation between the percentage of CD3 before treatment and ARR. This is also matched with the increase in the percentage of CD3 after immunotherapy and in the remission period. This can assist clinical work in assessing disease prognosis. However, our sample size is small, and we need to expand the sample for further prospective studies.

Immunoglobulin and complement components are deposited in the center of blood vessels in NMOSD lesions [40]. Complement is considered to be an important mediator of natural immune defense and inflammation. Complement activation is an important mechanism for the pathogenesis of NMOSD. Serum immunoglobulin and complement levels can be used as indicators to evaluate different stages and severities of NMOSD [17]. Studies have reported that the EDSS score is significantly positively correlated with IgG [17]. Chen et al. found that in the acute phase (initial course < 1 month), NMOSD patients had increased peripheral blood IgG, and AQP4-IgG-positive patients had a higher IgG [17]. In this study, a higher IgG was also found in patients at the acute phase, indicating that IgG was involved in the immune defense process at the acute phase, but no difference was found between AQP4 antibody-negative and AQP4 antibody-positive IgG. AQP4-IgG is closely related to immunoglobulin, and AQP4-IgG belongs to the IgG1 homotype and is the most effective immunoglobulin subclass to activate the complement system [41]. This study showed that IgG was lower after immunotherapy and that immunotherapy can inhibit IgG. It has been reported that elimination of IgG through plasma exchange can effectively reduce the acute symptoms of patients with NMOSD [42]. Some studies reported a positive correlation between serum C3 lysis product C3a and EDSS score [43]. Another study showed that CH50 is reduced in NMOSD patients, and there was no difference between C3 and C4 in disease stage and AQP4-IgG status. Other studies have reported that serum total complement activity (CH50) levels increase in positive patients during relapse [44]. One study showed that there was no correlation between serum C3 and C4 levels in NMOSD patients and EDSS scores [18]. As acute reactive proteins, C3 and C4 are synthesized in the acute phase and will activate and deplete complement in the onset of NMOSD. There is a balance between the two. According to multiple factors, we found in the regression model that age, sex, spinal cord-involved segment, and C3 were influencing factors for severe motor disability, while age, male sex, and spinal cord-involved long segment had previously been proven to be risk factors for poor prognosis [45,46]. It also confirmed previous studies that C3 is involved in the pathogenesis of NMOSD.

Hakobyan et al. found that the combination of plasma C1-inhibitor (C1 inh) and terminal complement complex (TCC) had good predictive value in distinguishing NMOSD from MS (AUC = 0.98), while C1inh and C5 can distinguish NMOSD from controls (AUC: 0.94) [47]. Qin et al. found that a serum C3 and C4 combined prediction model used to distinguish AQP4-IgG-positive and MOG-IgG-positive patients has moderate predictability (AUC = 0.787) [48]. Previous studies have shown that complement is highly predictive of NMOSD research. Our ROC curve analysis showed that the increase in peripheral blood complement C3 and C4 was a reference index to predict the occurrence of severe motor disability. Therefore, complement protein may be a diagnostic marker of NMOSD, and complement C3 and C4 may contribute to the early detection of NMOSD patients with severe motor disability, thereby improving the prognosis of these patients. However, the predictive value is weak, and the sample size needs to be further expanded to continue research.

This study has some limitations. First, this study is a retrospective study with certain biases. Second, because it is a study of commonly used clinical hospitalization indicators, there is no further classification study on the many subgroups of CD4+ T cells and CD8+ T cells. Third, there was no blood sample collection or immune indicator tracking for patients at multiple time points and larger sample sizes after treatment. Fourth, longitudinal studies have a small sample size, and further expansion of the sample size is needed for prospective follow-up studies.

## 5. Conclusions

This study demonstrates the role of peripheral blood T lymphocytes, complement, and immunoglobulin in the inflammatory response, suggesting a correlation between clinical immune indicators and disease activity, age, and immunotherapy. In addition, age, sex, spinal cord involvement, and serum C3 are influencing factors for severe motor disability. Peripheral blood C3 and C4 are the reference indicators for predicting severe motor disability.

Peripheral blood T lymphocytes, complement, and immunoglobulin are convenient and routine clinical indicators that are convenient for implementation in clinical work. They have a certain reference value for disease staging, recurrence, drug efficacy, and motor disability and improve our understanding of clinical immune indicators in NMOSD patients, but whether they can be used as biomarkers for clinical prognosis remains to be further studied.

## Figures and Tables

**Table 1:** Characteristics of all patients with NMOSD in the analysis.

Characteristic	NMOSD (*n* = 258)
Age at the time of blood collection, years (mean ± SD)	44.1 ± 14.5
Female, *n* (%)	233 (90.3%)
Age at onset, years, (mean ± SD)	40.2 ± 14.7
AQP4-IgG positive, *n* (%)	221 (85.7%)
Disease duration, years, IQR	1.42 (5.33)
ARR, IQR	0.43 (1.09)
EDSS score at the time of blood collection, IQR	3.0 (4.0)
EDSS score at last follow-up, IQR	3.0 (3.0)
Follow-up time, months, IQR	24.5 (25.3)
Initial onset syndromes, *n* (%)	
Optic neuritis	85 (32.9)
Transverse myelitis	105 (40.7)
Area postrema syndrome	11 (4.3)
Brain stem syndrome	7 (2.7)
Cerebral syndrome	2 (0.8)
ON + TM	18 (7.0)
Multifocal *	30 (11.6)
Disease staging, *n* (%)	
Acute phase	168 (65.1%)
Remission phase	90 (34.9%)
Treatment, *n* (%)	
No	107 (41.5%)
Yes	151 (58.5%)
Glucocorticoid only	63 (41.7%)
Immunosuppressant only	23 (15.2%)
Glucocorticoid and immunosuppressants	65 (43.0%)
Immunosuppressant, *n* (%)	
MMF	62 (41.3%)
RTX	14 (9.3%)
AZA	11 (7.3%)
CTX	1 (0.7%)
Treatment time, months, IQR	6.5 (23.0)
Relapse after treatment, *n* (%)	
Yes	116 (76.8%)
No	35 (23.2%)

Values are n (%), mean ± SD, or median (interquartile range). NMOSD: neuromyelitis optica spectrum disorders; ARR: annualized relapse rate; EDSS: expanded disability status scale; IQR: interquartile range; SD: standard deviation; HCs: healthy controls; MMF: mycophenolate mofetil; AZA: azathioprine; RTX: rituximab; CTX: cyclophosphamide. * Two or more initial clinical syndromes.

**Table 2:** Comparison of T lymphocyte subsets, complement, and immunoglobulinbetween NMOSD patients and HCs.

Characteristic	NMOSD (*n* = 258)	HCs (*n* = 200)	*p* Value
Age at the time of blood collection, years (mean ± SD)	44.1 ± 14.5	39.6 ± 15.2	0.557
Female, *n* (%)	233 (90.3%)	168 (84.0%)	0.594
T lymphocyte subsets			
CD3%	71.4 (13.8)	73.8 (9.5)	0.013 **
CD4%	38.8 (12.8)	42.2 (9.1)	<0.001 ***
CD8%	26.4 (11.6)	25.8 (9.7)	0.096
CD4/CD8	1.43 (0.87)	1.66 (0.86)	<0.001 ***
Complement C3 (g/L)	0.829 (0.215)	0.844 (0.187)	0.285
Complement C4 (g/L)	0.177 (0.094)	0.221 (0.084)	<0.001 **
IgG (g/L)	10.95 (4.49)	11.80 (3.05)	0.026 *

Values are n (%), mean ± SD, or median (interquartile range). NMOSD: neuromyelitis optica spectrum disorders; SD: standard deviation; HCs: healthy controls; normal range of CD3 cell subset ratio: 66.9–83.1%; normal range of CD4 cell subset ratio: 33.19–47.85%; normal range of CD8 cell subset ratio: 20.4–34.7%; normal range of CD4/CD8 ratio: 0.97–2.31; C3: 0.785–1.520 g/L; normal range of complement C4: 0.145–0.360 g/L; normal range of immunoglobulin: 8–15.5 g/L. * *p* < 0.05; ** *p* < 0.01; *** *p* < 0.001.

**Table 3:** Multiple linear regression of immune indicators in NMOSD patients.

	B	Standard Error	*p* Value
CD3% (R^2^ = 0.058)			
Age at the time of blood collection, years	−0.122	0.047	0.010
Disease stage	4.504	1.436	0.002
Acute phase			
Remission phase	1		
CD8% (R^2^ = 0.091)			
Age at the time of blood collection, years	−0.128	−0.037	0.001
Disease stage	3.300	1.128	0.004
Acute phase			
Remission phase	1		
Treatment	2.361	1.092	0.032
No			
Yes	1		
CD4/CD8 (R^2^ = 0.075)			
Age at the time of blood collection, years	0.011	0.003	0.000
Treatment	−0.224	0.087	0.010
No			
Yes	1		
C4 (R^2^ = 0.038)			
Gender	−0.036	0.012	0.003
Male			
Female	1		
Age at the time of blood collection, years	0.001	0.000	0.012
IgG (R^2^ = 0.099)			
Disease stage	−1.981	0.604	0.001
Acute phase			
Remission phase	1		
Treatment	−1.896	0.570	0.001
No			
Yes	1		

**Table 4:** Comparison of T lymphocyte subgroups in NMOSD patients receiving different immunosuppressive treatments.

Treatment Drugs	CD3%	CD4%	CD8%	CD4/CD8 Ratio
MMF (*n* = 62)	68.5 (16.7)	35.7 (16.9)	26.0 (11.3)	1.30 (0.92)
RTX (*n* = 14)	78.3 (9.0) ^a^	41.9 (13.4)	28.3 (12.2) ^b^	1.36 (0.81)
AZA (*n* = 11)	81.9 (22.0)	40.7 (16.5)	36.400 (14.7)	1.15 (0.81)
*p* value	0.004	0.107	0.049	0.667

^a^ After Bonferroni adjustment, compared with the group receiving MMF, *p* = 0.012; ^b^ After Bonferroni adjustment, compared with the group receiving MMF, *p* = 0.042; MMF: mycophenolate mofetil; AZA: azathioprine; RTX: rituximab.

**Table 5:** Comparison of complement and IgG in NMOSD patients receiving different immunosuppressive treatments.

Treatment Drugs	Complement C3 (g/L)	Complement C4 (g/L)	IgG (g/L)
MMF (*n* = 62)	0.837 (0.228)	0.189 (0.098)	10.35 (5.06)
RTX (*n* = 14)	0.788 (0.324)	0.165 (0.068)	9.05 (3.16)
AZA (*n* = 11)	0.760 (0.219)	0.158 (0.060)	9.98 (3.84)
*p* value	0.623	0.568	0.188

MMF: mycophenolate mofetil; AZA: azathioprine; RTX: rituximab.

**Table 6:** Binary logistic regression multivariate analysis of severe motor disability.

Variable	OR (95% CI)	*p* Value
Blood collection age	1.080 (1.044–1.117)	0.000
Gender		
Female	1	
Male	0.170 (0.041–0.701)	0.014
Involved segment of spinal cord		
≥3	1	
<3	7.763 (2.202–27.370)	0.001
Complement C3	22.598 (2.532–201.647)	0.005

OR: odds ratio; 95% CI: 95% confidence interval.

## Data Availability

Not applicable.

## Abbreviations

annualized relapse rate	ARR
aquaporin-4	AQP4-IgG
azathioprine	AZA
central nervous system	CNS
expanded disability status scale	EDSS
follicular helper T cell	Tfh
healthy controls	HCs
rituximab	RTX
regulatory T cells	Treg
mycophenolate mofetil	MMF
neuromyelitis optica spectrum disorders	NMOSD
